# On the Use of Muriate of Barytes in Scrofulous Affections

**Published:** 1846-12

**Authors:** A. J. Walshe


### SELECTIONS

FROM

### AMERICAN AND FOREIGN JOURNALS.

---

*On the Use of Muriate of Barytes in the Treatment of Scrofulous Affections.* By A. J. Walshe, M.D., F.R.C.S.I., &c. Dr. Adair Crawford was the first to call the attention of the profession to the use of this remedial agent in the treatment of scrofulous affections. There is a notice of it by him in the Medical Communications for the year 1789. He remarked that it combined the action of an evacuant, a deobstruent, and a tonic; that its incautious use was likely to be attended with a host of unpleasant and even dangerous symptoms, as loss of appetite, thirst, nausea, constipation, deafness, loss of sight, and paralysis of the voluntary muscles; to obviate this, he advises the use of a saturated solution, gradually increased from four to ten drops, as a dose.

He dissolved half a drachm in an ounce of distilled water, and gave from ten to fifty drops, according to age—that is, roughly from half a grain to three grains of the solid muriate. When he administered it to infants, he added a syrup to diminish its irritant effects; when the stomach was affected with spasm, he combined it with some aromatic or antispasmodic; he mentions that its activity in certain diseases of the skin is much increased by the addition of emetic wine, Plummer's pill and cicuta. In very delicate subjects, he exhibited it according to the following formula:—Muriate of barytes, muriate of iron, of each half a drachm. Water distilled, syrup of orange peel, of each an ounce. The dose of which is from twenty to thirty drops every three hours. Dr. Crawford has also recommended its conjunction with iron; indeeed, the solution of barytes, which he used in his first experiments, contained a minute proportion of iron.

The French physicians usually recommend a drachm of the salt to be dissolved in two pints of distilled water, of this a teaspoonful, that is about half a grain of the barytes is taken in a teacupful of infusion of hops, or some other bitter, every morning, fasting; this dose is gradually increased, its effect being watched with great care. The form in which I have usually exhibited it varies from that heretofore followed in being in pill. I at first exhibited it in this manner to avoid as much as possible the too speedy decomposition of the medicine in the stomach, and at the same time to prevent its being so rapidly absorbed as it would be in a fluid state. I have always commenced with very small quantities, one-twelfth of a grain three times a day, gradually increasing the frequency of the doses, rather than the quantity in each. I think it desirable that it should be taken after meals, so as to defend the stomach from its rapid action. With these precautions, though I must confess it was with some anxiety that I awaited its effects the first time, I have never experienced any inconvenience in its exhibition.

In that form of ophthalmia attended with great intolerance of light, so characteristic of the scrofulous form of this disease, Dr. Walshe has observed it very useful. He says, however,

I do not wish it to be understood as my opinion that muriate of barytes should supersede iodine in the treatment of scrofulous diseases, in which we frequently derive such eminent services from the latter, but I think I am warranted in inferring, after a careful examination of these cases, that there are some cases of scrofulous disease, in which iodine is useless, or may be worse than useless, and when we may derive considerable service from the muriate of barytes; and further, that there are some cases in which we experience beneficial results from iodine up to a certain point, beyond which, if it be persisted in, its beneficial action ceases, and it proves noxious. In these cases the treatment may be advantageously taken up with the barytes, and vice versa.—*Dublin Med. Press.—Braithwaite.*

